# Predictors of underlying neuroblastoma and neurodevelopmental outcomes in children with opsoclonus-myoclonus syndrome: a retrospective cohort study in China

**DOI:** 10.3389/fneur.2026.1892635

**Published:** 2026-10-01

**Authors:** Qing Sun, Penghui Wu, Yao Xie, Yuwu Jiang, Weihong Zhao

**Affiliations:** Department of Children's Medical Center, Peking University First Hospital, Beijing, China

**Keywords:** China, neuroblastoma, neurodevelopmental outcomes, opsoclonus-myoclonus syndrome, retrospective

## Abstract

**Aim:**

To explore whether distinct clinical features can serve as predictive indicators for the underlying NB and examine the neurodevelopmental outcomes in children diagnosed with opsoclonus-myoclonus syndrome (OMS).

**Methods:**

We retrospectively enrolled children with a definitive diagnosis of OMS at Peking University First Hospital between January 2010 to January 2025. Demographic data, clinical characteristics were collected to compare based on with or without tumor. Neurodevelopmental outcomes were classified based on adaptive behavior assessments.

**Results:**

Seventy-three children were totally included, including 30 with NB and 43 without NB. 52 children (71%) had neurological signs onset concentrated between 1 and 2 years of age. There was significant difference in the composition ratio of initial symptoms between the two groups of OMS patients. The proportion of opsoclonus as the initial symptom in the OMS with NB group (20%) was higher than that in the OMS without NB group (2%). 48 patients with neurological remission underwent assessment of adaptive behavior. By the last follow-up, 28 (58%) patients had good adaptive behavior, and 20 (42%) patients had poor adaptive behavior. The main variables influencing the neurodevelopmental prognosis in OMS patients, ranked from highest to lowest importance, are as follows: chemotherapy, rituximab, myoclonus at onset, immunotherapy initiation, tumor, age at onset, gender, ataxia at onset, OMS severity at onset, opsoclonus at onset, and ataxia at onset. The multivariate logistic regression model further confirmed that myoclonus at onset (OR = 14.33, 95% CI: 1.77–116.4, *p* = 0.01) was an independent risk factor for poor outcomes, whereas neuroblastoma (OR = 0.11, 95% CI: 0.02–0.68, *p* = 0.02) and chemotherapy (OR = 0.07, 95% CI: 0.01–0.58, *p* = 0.02) were independent protective factors. The ROC curve yielded an AUC of 0.856, indicating good discriminative ability of the model.

**Interpretation:**

Opsoclonus as the initial symptom may be a predictive indicator for the underlying NB. With NB and chemotherapy are the key factors for the favorable neurodevelopmental prognosis of OMS patients.

## Highlights

- Opsoclonus as the initial symptom may serve as a predictive indicator for tumor.
- With NB and chemotherapy are the key factors for the favorable neurodevelopmental prognosis of OMS patients.

## 1 Introduction

Opsoclonus-myoclonus syndrome (OMS) is a rare neuroimmune disorder, typically manifesting during the second year of life. The estimated incidence in pediatric populations ranges from 0.27 to 0.40 cases per million children (1). Clinically, OMS is characterized by a constellation of neurological symptoms, including cerebellar ataxia, opsoclonus—defined as involuntary, chaotic, high-frequency conjugate eye movements with horizontal, vertical, and torsional saccadic bursts, myoclonus, irritability, and behavioral abnormalities (including sleep disturbances) (2). No definitive disease-specific autoantibody has been consistently identified to date. Approximately 50% of the patients have an underlying neuroblastoma (NB). Due to the occult nature of NB, the majority of patients are initially diagnosed with OMS before the tumor is identified. It remains unclear whether distinct clinical features can serve as predictive indicators for the presence of underlying NB in OMS patients.

OMS demonstrates responsiveness to immunotherapy; however, it is frequently associated with high rates of relapse and long-term neurological sequelae (3, 4). Several studies suggest that initial combination therapy with intravenous immunoglobulin (IVIG), glucocorticoid (GC) and rituximab may be more effective in improving neurological outcomes. Some patients with NB also require risk-adapted chemotherapy. Nonetheless, the overall benefit of combination therapy remains under debate, and treatment strategies are often individualized based on disease severity.

Despite increasing recognition of OMS, epidemiologic data remain limited and inconsistent, mainly due to its rarity (4–8). To date, no large-scale cohort studies on OMS have been reported in mainland China (1, 9). In this study, we retrospectively analyzed a cohort of patients diagnosed with OMS at our center between January 2010 and January 2025, aimed to explore whether distinct clinical features can serve as predictive indicators for the underlying NB and elucidate the factors influencing neurodevelopmental prognosis in OMS children.

## 2 Patients and methods

### 2.1 Participants

This study was a single-center, retrospective study conducted at Peking University First Hospital. Inclusion criteria comprised patients between 0 and 18 years of age who had a confirmed diagnosis of OMS. Data were collected from January 2010 to January 2025. The diagnostic criteria for OMS were as follows (2): ① opsoclonus, ② myoclonus and/or ataxia, ③ behavioral abnormalities and/or sleep disturbances, ④ detection of neuroblastoma (NB); at least three of the above four items must be satisfied. OMS diagnosis verified by the first two authors. Exclusion criteria included an incorrect diagnosis of OMS occasioned by findings of nystagmus or unrelated eye movement abnormalities, verified seizures (not just bouts of myoclonus), acute cerebellar ataxia, or other neurological disorder. The definitive diagnosis of NB was established based on histopathological examination. Based on the presence or absence of associated NB, patients were classified into two groups: OMS with NB and OMS without NB.

### 2.2 Clinical data collection

We retrospectively collected demographic and clinical characteristics, including gender, date of birth, age at onset, age at OMS diagnosis, prodromal infection, neurological signs at onset, OMS severity at onset, laboratory test results, imaging examinations and treatment regimens. NB-related characteristics were also collected, encompassing tumor screening results, tumor location, histopathological subtype, disease stage, risk stratification, and recurrence status.

Patients were followed up regularly since 2019 until death or last follow-up, with September 30, 2025 serving as the endpoint of follow-up.

### 2.3 Neurological symptom

The Mitchell and Pike OMS rating scale (9) was used to evaluate the severity of neurological symptoms. This scale assesses neurological symptoms in patients with OMS from five aspects: stance, gait, hand and arm function, opsoclonus, and mood/behavior. For each subitem maximum score is 3 points, with 0 point indicating normal, 1 point indicating mild, 2 points indicating moderate, and 3 points indicating severe. This scale ranges from 0 to 15, with higher scores indicating greater symptom severity: scores of 0–5 denote mild disease, 6–10 moderate disease, and 11–15 severe disease. Remission was defined as follows: the Mitchell and Pike OMS score stabilized at ≤3 after 6 months of observation.

### 2.4 Neurodevelopmental outcome

Adaptive Behavior Assessment System, Second Edition (ABAS-II) was used to evaluate the neurodevelopmental status of the enrolled patients. According to the developmental status at the latest follow-up (patients with remission), patients were divided into two groups based on outcomes.

ABAS-II, with a total score of 120, is a rating scale that evaluates the skills an individual needs to perform daily activities effectively and independently at home, school, and in the community. A score of ≥90 was defined as a favorable prognosis, and a score of <90 was defined as a poor prognosis.

### 2.5 Statistical analysis

Statistical analyses were performed using SPSS version 20.0. Continuous variables were described using medians (ranges) and categorical variables were described using frequencies (percentages). Chi-square test was used to compare frequencies, and medians were compared using Kruskal–Wallis (K–W) tests. *P* values <0.05 were considered statistically significant.

To perform feature selection and data dimensionality reduction, a random forest algorithm was applied. The mean decrease in the Gini coefficient (MeanDecreaseGini) was used as the measure of variable importance, allowing for variable selection and importance ranking. For OMS children without NB, missing values for variables including chemotherapy and tumor were handled by listwise deletion.

Univariate and multivariate logistic regression analyses were performed to determine the direction and strength of associations between each predictor and adaptive behavior outcomes. Results were presented as odds ratio (OR) with 95% confidence interval (CI), and *p* < 0.05 was considered statistically significant. The discriminative ability of the model was evaluated using the receiver operating characteristic (ROC) curve, and the area under the curve (AUC) with its 95% CI was calculated.

The retrospective observation study was approved by the Ethics Committee of Peking University First Hospital (Approval No. 2023-233-003). Due attention was paid to non-identifiability.

## 3 Results

### 3.1 Demographic information

A total of seventy-three patients with OMS were enrolled in this study, comprising thirty patients with NB and forty-three patients without NB. Of the seventy-three patients, forty-four (60%) were male and twenty-nine (40%) were female. The median age at onset of neurological symptoms was 1.58 (0.58–4.50) years. 52 children (71%) had neurological signs onset concentrated between 1 and 2 years of age, which is also the highest prevalence of neurological signs onset for OMS symptoms. Among them, 21 children (21/30, 70%) in the OMS with neuroblastoma (NB) group and 31 children (31/43, 72.1%) in the OMS without NB group developed the disease within this age range (Figure 1).

**Figure 1:**
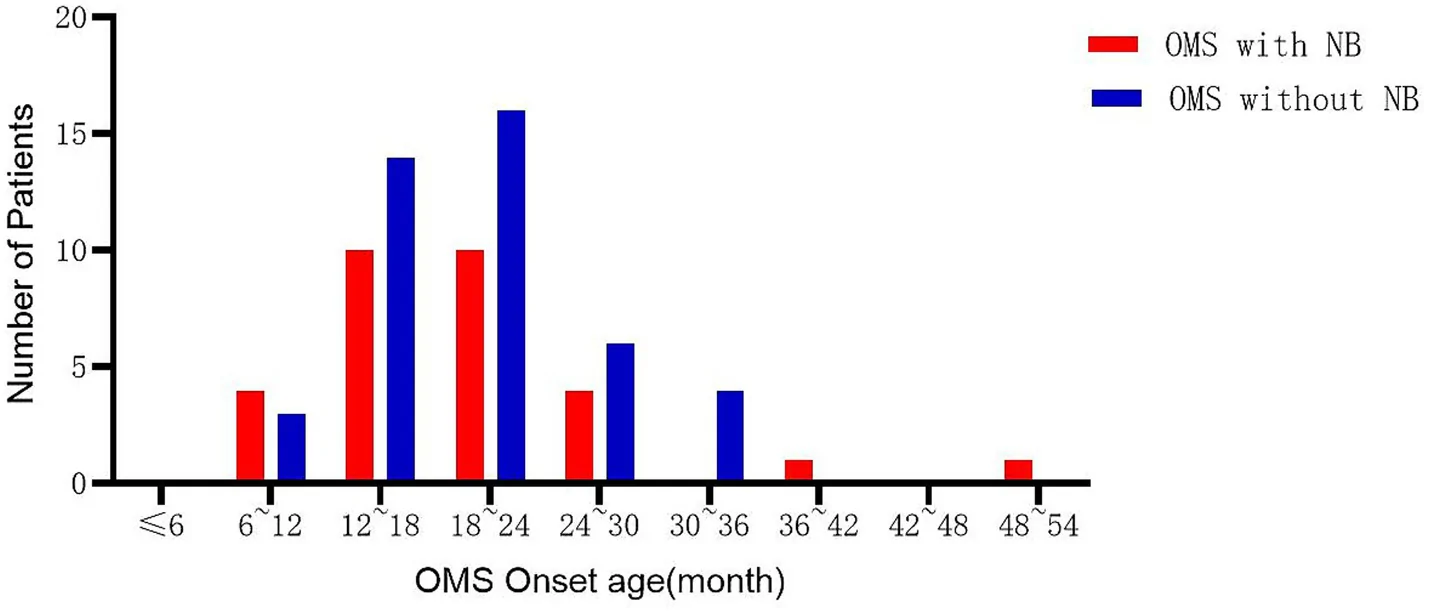
Prevalence of OMS by age of onset for patients with and without NB.

The median age at onset of neurological symptoms was well balanced between the two groups, indicating no significant difference in age distribution. Similarly, there was no statistically significant difference in gender distribution, although a trend toward greater male predominance was observed in the OMS with NB group (Table 1).

**Table 1:** Comparison of demographic and clinical features of OMS based on tumor detection.

OMS feature	OMS with NB (*N* = 30)	OMS without NB (*N* = 43)	*P* value
Age of onset, years	1.58 (0.58–4.50)	1.58 (0.67–2.75)	0.929
Male	20 (67%)	24 (56%)	0.351
Prodromal infection	11 (37%)	22 (51%)	0.221
Neurological signs at onset			0.020
Opsoclonus	6 (20%)	1 (2%)	
Myoclonus	4 (13%)	11 (26%)	
Ataxia	20 (67%)	27 (63%)	
Behavioral abnormalities	0	4 (9%)	
OMS severity at onset			0.371
Mild (0–5)	8 (26%)	9 (20%)	
Moderate (6–10)	8 (26%)	17 (40%)	
Severe (11–15)	14 (47%)	17 (40%)	
Time to OMS diagnosis, years	0.25 (0.08–1.33)	0.33 (0.08–1.83)	0.703
Cerebrospinal fluid	*N* = 12	*N* = 9	
Leukocytes (/mm^3^)	2 (0–8)	2 (0–6)	0.845
OCB-positive	0	1 (11.1%)	0.428

### 3.2 Clinical characteristics of OMS with or without NB

The clinical characteristics of OMS patients with and without NB are summarized in Table 1. Parent-reported histories of prodromal infections were evaluated, which were present in 37% (*n* = 11) of the OMS with NB group and 51% (*n* = 22) of the OMS without NB group. No significant difference was found in the proportion of prodromal infections between the two groups. Although some patients underwent testing for a range of infectious agents, no consistent or specific infectious etiology was identified through clinical or laboratory assessments.

There was significant difference in the composition ratio of initial symptoms between the two groups of OMS patients. Although the most common initial symptom in both groups was ataxia, OMS patients with NB had a higher proportion of opsoclonus as the initial symptom, while OMS patients without NB had a higher proportion of myoclonus and behavioral abnormalities as the initial symptoms. It is worth noting that, four children (9%) in the OMS without NB group presented with behavioral changes as the initial symptoms, whereas no child in OMS with NB group had this symptom as the initial symptom.

All patients’ OMS severity scores at onset (within 2 weeks of neurological symptom onset) were collected. No statistically significant differences in OMS severity during the early stage were observed between the two groups.

The interval from the onset of OMS diagnosis, which exhibited considerable variability across the cohort, did not differ significantly between the two groups. A total of 49% (*n* = 36) patients were initially misdiagnosed with other neurological conditions prior to the correct diagnosis of OMS. Among these, 26% (*n* = 19) cases were misdiagnosed as postinfectious cerebellar ataxia during the early clinical course.

Cerebrospinal fluid (CSF) analysis revealed minimal elevation in white blood cell (WBC) counts in both OMS subgroups, with all values ≤8/mm^3^. Intrathecal oligoclonal band (OCB) production was detected in only one patient without NB. All 73 patients underwent cranial MRI at onset. Imaging reports were summarized for 28 patients whose original images were unavailable, while the original imaging data were re-analyzed for 45 patients whose original images could be obtained. Cranial MRI findings were normal in all patients.

### 3.3 Tumor characteristics

All seventy-three patients were screened for tumors using NB-specific biomarkers and contrast-enhanced computed tomography (CT) imaging. Ultimately, NB was detected in 30 OMS patients. The median interval from neurological signs onset to detection of NB was 3 (0.5–21)months. Notably, two patients showed no evidence of tumors on initial contrast-enhanced CT scans; however, follow-up imaging performed 3 and 14 months later, respectively, revealed the presence of tumors.

When the tumor was first detected, tumor location, histopathological classification, and disease stage according to the International Neuroblastoma Risk Group Staging System (INRGSS) are summarized in Table 2. In the OMS with NB group, the most common tumor locations were adrenal and retroperitoneal sites, followed by the mediastinum; pelvic involvement was rare. Histopathologically, neuroblastoma (*n* = 15, 50%) and ganglioneuroma (*n* = 13, 43%) were the predominant subtypes. No patient exhibited distant metastasis at the time of initial tumor diagnosis. According to the INRGSS, 57% (*n* = 17) of patients were classified as stage L1, with 43% (*n* = 13) of patients were categorized as stage L2. According to the Children’s Oncology Group (COG) risk classification system, 30% (*n* = 9) patients were classified as very low risk, 57% (*n* = 17) as low risk, 10% (*n* = 3) as intermediate risk, and 3% (*n* = 1) as high risk due to *MYCN* amplification.

**Table 2:** Tumor characteristics in OMS with NB group.

Tumor feature	*N*	%
Tumor location		
Adrenal gland	11	36.7
Retroperitoneum (except adrenal gland)	11	36.7
Mediastinum	5	16.7
Pelvis	3	10.0
Tumor type		
GN	2	6.7
GNB, nodular	8	26.7
GNB, intermixed	5	16.7
NB	15	50.0
INRGSS		
L1	17	56.7
L2	13	43.3
M/MS	0	0
Risk classification		
Very low risk	9	30.0
Low risk	17	56.7
Intermediate risk	3	10.0
High risk	1	3.3

NB, neuroblastoma; GNB, ganglioneuroblastoma; GN, ganglioneuroma.

### 3.4 Treatment

Immunotherapy serves as the primary therapeutic approach for OMS. Patients without NB received immunotherapy alone, whereas those with NB were managed with a multimodal regimen that included immunotherapy, risk-adapted chemotherapy, surgical resection, and/or radiotherapy (Table 3).

**Table 3:** Treatment practice patterns in OMS patients with and without NB.

Treatment practice patterns	OMS with NB (*N* = 30)	OMS without NB (*N* = 43)
Surgery	30 (100.0%)	–
Chemotherapy	24 (80.0%)	–
Combined with GC and IVIG	8 (26.7%)	–
Combined with GC, IVIG and rituximab	2 (6.7%)	–
Immunotherapy	6 (20.0%)	43 (100.0%)
GC and IVIG	5 (16.7%)	27 (62.8%)
GC, IVIG and rituximab	1 (3.3%)	16 (37.2%)
Complete resolution of OMS symptoms	*N* = 28	*N* = 35
Duration of OMS symptoms, months	10 (3–32)	15 (6–76)

In this study, immunotherapy was classified into two patterns. Before 2015, inmunotherapy consisted of GC and IVIG. Since 2015, rituximab has been incorporated into the treatment protocol along with GC and IVIG at our institution.

In the OMS with NB group, all patients underwent tumor resection. 24 patients (80%) received risk-adapted chemotherapy. 10 patients (33%) received immunotherapy either before or after chemotherapy, and among these, 2 patients were treated with rituximab. 6 patients (20%) received immunotherapy without chemotherapy, with 1 patients received rituximab. The median interval from the OMS onset to the initiation of immunotherapy or chemotherapy among the 30 patients was 1 (0.5–17) months.

In OMS without NB group, all patients received immunotherapy. 27 patients (63%) received GC and IVIG alone, and 16 patients (37%) received rituximab in addition to the GC and IVIG treatment. The median time from OMS onset to initiation of immunotherapy among the 43 patients was 1 (0.5–22) months.

### 3.5 Prognosis

By September 2025, only one patient died due to tumor recurrence. Among survivors, 63 patients achieved neurological remission. 28 patients belonged to the OMS with NB group, and 35 patients belonged to the OMS without NB group. The median duration of neurological symptoms was 10 (3–32) months in patients with NB and 15 (6–76) months in those without NB. The difference in symptom duration between the two groups was statistically significant (*p* = 0.007) (Table 3).

During follow-up, 48 patients with neurological remission underwent assessment of adaptive behavior. The median follow-up duration was 8.0 (1.0–13.5) years. Among these patients, 23 (48%) belonged to the OMS with NB group, and 25 (52%) were classified in the OMS without NB group. By the last follow-up, 28 (58%) patients had good adaptive behavior, and 20 (42%) patients had poor adaptive behavior.

The adaptive behavior status of 48 patients were used as the dependent variable. Eleven variables related to the demographic characteristics, clinical manifestations, diagnosis and treatment information were included in a random forest model, with the number of trees set to 500 and the random seed set to 123,456. The results showed that the variables influencing the prognosis of adaptive behavior in OMS patients, in descending order of importance, were: chemotherapy, rituximab, myoclonus at onset, immunotherapy initiation, tumor, age at onset, gender, ataxia at onset, OMS severity at onset, opsoclonus at onset, and behavioral abnormalities at onset (Figure 2).

**Figure 2:**
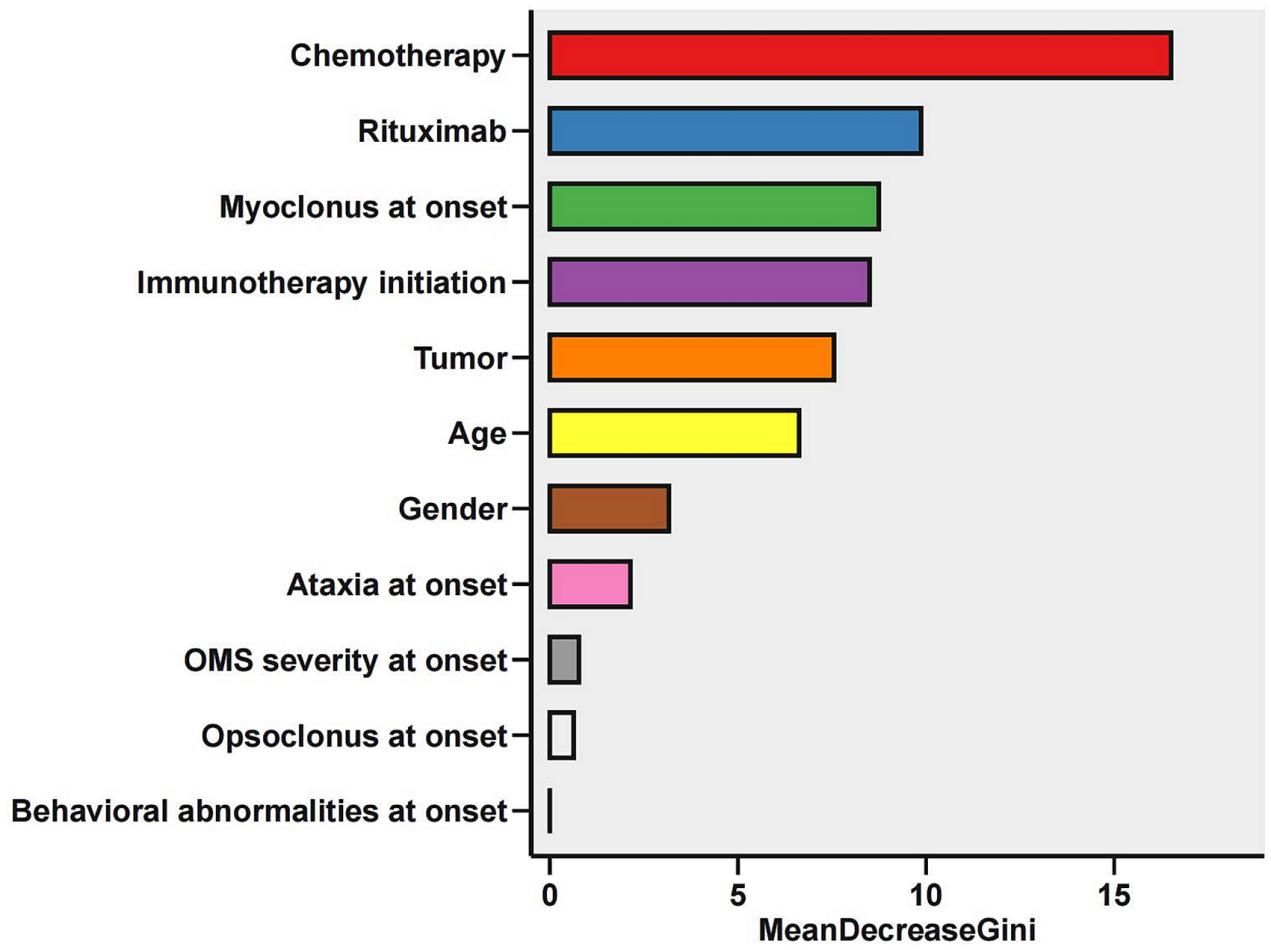
The importance ranking of the 11 variables affecting adaptive behavior outcome.

We further performed logistic regression analyses to clarify the direction and strength of each variable. Univariate logistic regression analyses identified age at onset (OR = 1.08, 95% CI: 1.00–1.16, *p* = 0.04), myoclonus as onset (OR = 7.27, 95% CI: 1.68–31.5, *p* = 0.008), and immunotherapy initiation (OR = 1.34, 95% CI: 1.02–1.76, *p* = 0.03) as significant risk factors for unfavorable outcomes, while tumor (OR = 0.22, 95% CI: 0.07–0.75, *p* = 0.02) and chemotherapy (OR = 0.14, 95% CI: 0.03–0.56, *p* = 0.006) were protective factors. Opsoclonus at onset (OR = 0.17, 95% CI: 0.01–1.36, *p* = 0.09) showed a protective trend without statistical significance (Figure 3). Variables with *p* < 0.10 were entered into multivariate logistic regression with backward stepwise selection. The final model identified myoclonus at onset (OR = 14.33, 95% CI: 1.77–116.4, *p* = 0.01) as an independent risk factor, and tumor (OR = 0.11, 95% CI: 0.02–0.68, *p* = 0.02) and chemotherapy (OR = 0.07, 95% CI: 0.01–0.58, *p* = 0.02) as independent protective factors for favorable outcomes (Figure 4). The ROC curve yielded an AUC of 0.856 (95% CI, 0.742–0.970), indicating good discriminative ability of the model (Figure 5).

**Figure 3:**
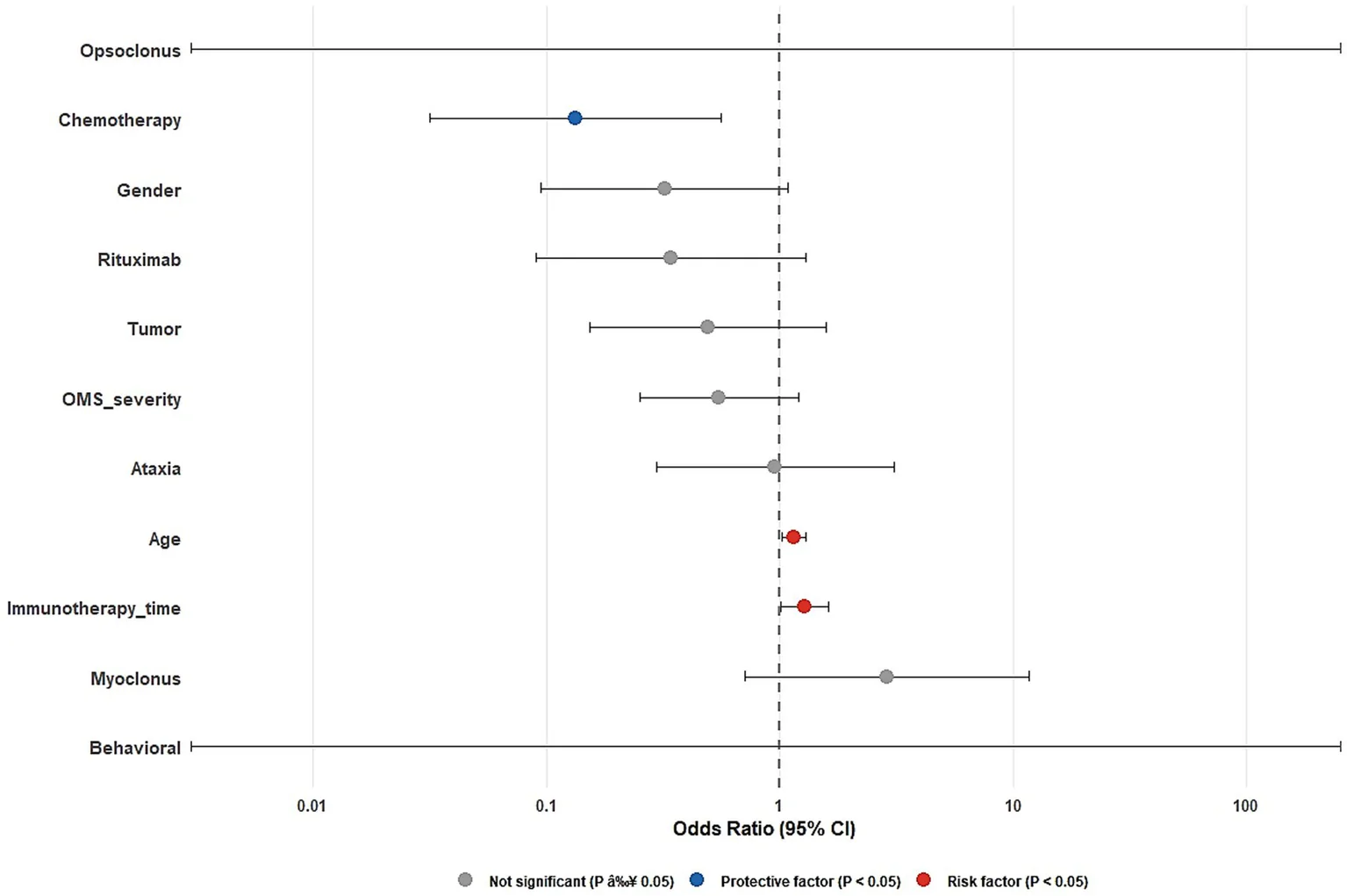
Univariate logistic regression analysis of 11 variables.

**Figure 4:**
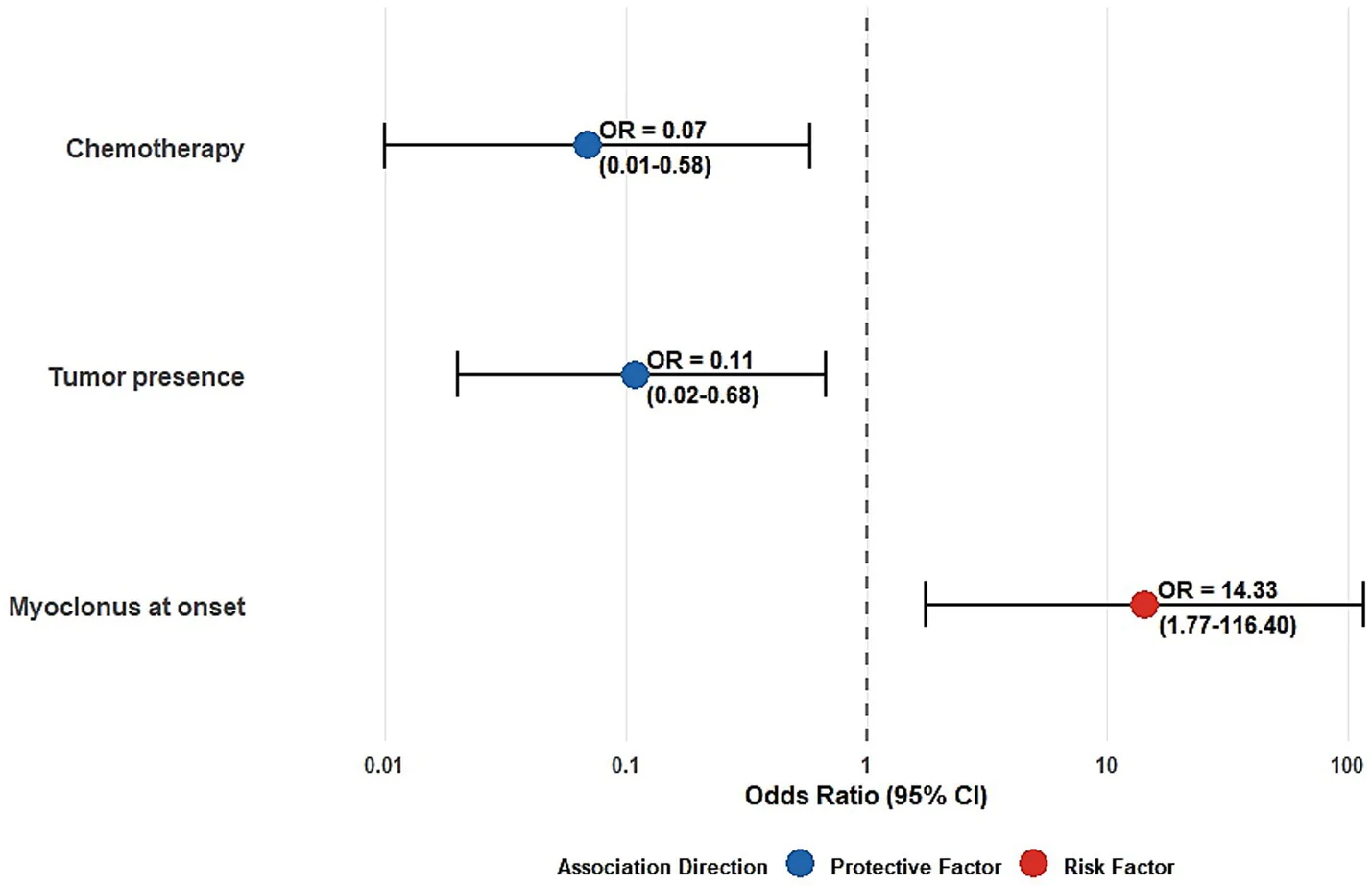
Multivariate logistic regression model.

**Figure 5:**
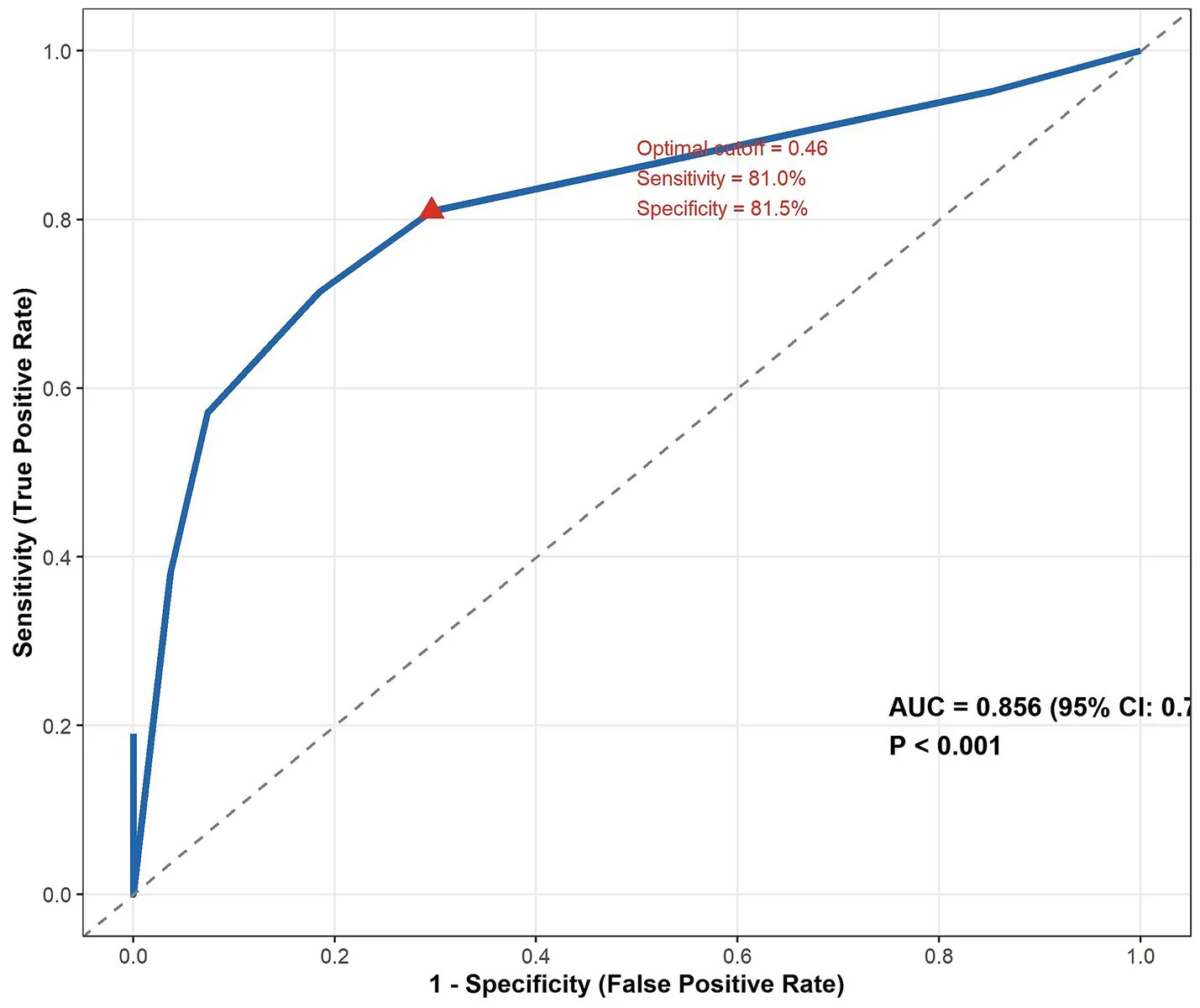
ROC curve for predicting unfavorable outcomes.

## 4 Discussion

This study retrospectively collected clinical data from pediatric-onset OMS patients treated at a single center in China. To date, this report presents the largest cohort of pediatric-onset OMS cases in China. The study systematically compares the clinical features of OMS patients with and without NB, aiming to analyze and summarize the distinctive clinical characteristics of these subgroups and elucidate the factors influencing neurodevelopmental prognosis in OMS children. The findings may provide predictive indicators for the underlying NB and provide evidence-based insights for clinical management decisions.

The primary demographic observation was that OMS predominantly affects young children, with the highest incidence occurring between 1 and 2 years of age. In this cohort of 73 patients with OMS, the median age at onset of neurological symptoms was 18.5 (7–54) months, consistent with a peak incidence during the second year of life. Notably, a male predominance was observed in the present study, which contrasts with previous reports from the United Kingdom and the United States (4, 7, 10, 11). Whether this observed gender disparity reflects underlying differences in susceptibility related to ethnic, genetic, or geographic factors remains unclear and warrants further investigation through large-scale, multicenter studies.

The classical clinical manifestations of OMS include opsoclonus, myoclonus, ataxia, sleep disturbances and/or behavioral abnormalities. However, these hallmark features may not all be present simultaneously at disease onset (12). There was significant difference in the composition ratio of initial symptoms between the two groups of OMS patients. Although the most common initial symptom in both groups was ataxia, OMS patients with NB had a higher proportion of opsoclonus as the initial symptom, while OMS patients without NB had a higher proportion of myoclonus and behavioral abnormalities as the initial symptoms. This suggests that opsoclonus as the initial symptom may be a predictive indicator for the underlying NB. Notably, all patients who presented with isolated behavioral abnormalities as the initial symptom (*n* = 4, 9%) were in the OMS without NB group, suggesting that this clinical feature may serve as a potential negative predictor for underlying NB, although the small sample size precludes definitive conclusions. This observation raises the hypothesis that young children presenting with prominent behavioral changes or mood disturbances, in the absence of typical opsoclonus or myoclonus, may have a lower likelihood of harboring an underlying tumor. Future large-scale studies are warranted to validate the negative predictive value of behavioral abnormalities at onset, which could help refine tumor screening strategies and reduce unnecessary investigations in this subgroup.

In this study, ataxia was identified as the most common initial presenting symptom in both groups. This finding is consistent with previous reports: a Japanese case series identified opsoclonus and ataxia as the predominant symptoms (1), and a multicenter, multinational study of 389 pediatric cases also reported ataxia as the most frequent initial manifestation in children with OMS (7). The earlier emergence of ataxia compared to opsoclonus or myoclonus in the disease course may partially explain why OMS is frequently misdiagnosed as acute cerebellar ataxia. Indeed, patients with acute cerebellar ataxia often have a history of preceding viral infections—such as varicella—and commonly present with gait instability and ocular motor abnormalities (13), overlapping significantly with early features of OMS. However, abnormal eye movements in acute cerebellar ataxia predominantly manifest as horizontal nystagmus, characterized by rhythmic, biphasic oscillations of the eyes that typically resolve during sleep (14). In contrast, opsoclonus in OMS is defined by rapid, high-amplitude, multidirectional, and chaotic saccadic intrusions that occur involuntarily in both eyes, most prominently evident during attempted saccades (15). These abnormal eye movements may diminish slightly during visual fixation but persist during eye closure and, notably, continue during sleep—distinguishing them from nystagmus associated with cerebellar dysfunction.

Regarding CSF analysis, findings from Japanese literature indicate that the WBC in the CSF of OMS patients is typically normal or only mildly elevated—a result consistent with our study (1). With respect to CSF-OCB, data from the United States report a positivity rate of 36% (75/208) (5). In contrast, our study observed a considerably lower positivity rate of 4.8% (1/21). In this study, some patients showed no abnormalities on early cranial MRI, but longitudinal neuroimaging data during follow-up were unavailable. A case report described severe cerebellar atrophy on MRI and marked hypoperfusion on perfusion SPECT in a child with NB-OMS, suggesting that neuroimaging may serve as a non-invasive biomarker for disease severity and management in OMS (16).

Due to the occult nature and often small size of NB, the majority of patients are initially diagnosed with OMS before the tumor is identified. Current screening protocols for NB in OMS patients typically involve a combination of anatomical imaging—most commonly contrast-enhanced CT—and biochemical tumor marker assessment. Notably, two patients initially had negative findings on both contrast-enhanced CT, but repeat imaging subsequently revealed primary NB. These false-negative results were likely due to the small size of the tumors at initial evaluation, which limited their radiological detectability. This observation underscores the limitations of single-time-point screening and highlights the importance of longitudinal surveillance. Critically, even in OMS patients without an initially detectable NB, ongoing and periodic tumor screening is essential to reduce the risk of missed or delayed diagnoses.

This study further examined the clinicopathological characteristics of NB in patients with OMS. The results demonstrated that the primary tumor sites were predominantly located within the abdominal cavity, including the adrenal gland. Histopathologically, the majority of cases were classified as neuroblastoma, with minimal representation of other related entities such as ganglioneuroblastoma. According to the INRGSS, most tumors were categorized as stage L1 or L2, indicating localized disease. Risk group stratification revealed that the majority of patients fell into the very low-risk or low-risk categories. These findings are consistent with a favorable long-term prognosis and are associated with an extremely low mortality rate attributable to NB. Previous studies have consistently demonstrated that NB occurring in patients with OMS is typically associated with favorable biological characteristics (17–19). These clinical and biological features are in accordance with the findings observed in the present study, further supporting the notion that OMS-associated NB represents a distinct subgroup with inherently less aggressive tumor biology.

For OMS patients with NB, surgical intervention is mandatory. The primary therapeutic focus is on managing the underlying NB, with treatment strategies determined according to the risk stratification of the tumor (20–22). From the standpoint of OMS management, complete resection of the tumor alone is insufficient to achieve full resolution of neurological symptoms. This observation suggests that neurological damage in OMS is not directly caused by the tumor itself, but rather by a tumor-triggered, sustained autoimmune response (23, 24). Therefore, even after tumor eradication, neurological symptoms may persist or relapse without timely and adequate immunomodulatory intervention. Additional multimodal therapies, including chemotherapy and immunotherapy, are typically required to control the neurological symptoms.

For OMS patients without NB, immunotherapy constitutes the mainstay of treatment. A commonly employed therapeutic strategy includes the use of IVIG and GC, often in conjunction with second-line agents such as rituximab or cyclophosphamide (9). Prior small-scale retrospective studies have indicated that the addition of rituximab may be associated with improved neurologic recovery and reduced relapse rates in this population (25, 26). But there remains a lack of consensus on the optimal immunotherapeutic protocol for children with OMS, owing to limited high-quality prospective data.

In this study, the incidence of poor adaptive behavior among 48 patients with OMS was 42%. Eleven variables related to demographic characteristics, clinical manifestations, and diagnostic and treatment information of the OMS patients were included in a random forest model for importance ranking. The results showed that the most important variables affecting the prognosis of adaptive behavior in OMS patients were chemotherapy and rituximab. Chemotherapy, like rituximab, is essentially a multi-drug combination immunotherapy. Therefore, multi-drug combination immunotherapy is the key to improving the neurodevelopmental prognosis of OMS patients.

This study has several limitations. First, due to the long time span of this study, follow-up data were incomplete for a subset of children, potentially affecting interpretation. Second, given that chemotherapy was predominantly administered to patients in the OMS with NB group, the high importance of chemotherapy as a predictive variable may partly reflect the combined effect of tumor presence and more intensive immunosuppressive therapy on prognosis. The independent effect of chemotherapy per se warrants further validation in prospective studies with larger sample sizes. Thirdly, various developmental assessment scales such as Wechsler Intelligence Scale, were not used, potentially limiting the comprehensiveness of the neurodevelopmental outcome. Fourth, the limited sample size (*n* = 48) available for the prognostic analysis, combined with the relatively large number of predictors (*n* = 11) included in the random forest model, may have increased the risk of overfitting, potentially affecting the stability of the variable importance ranking. External validation in multicenter prospective cohorts is needed to confirm our findings. Future multicenter prospective cohort studies incorporating multiple developmental scales are needed to better optimize the treatment strategies for children with OMS.

## 5 Conclusion

In summary, by comparing the clinical characteristics of the two OMS groups, this study revealed that opsoclonus as the initial symptom may be a predictive indicator for the underlying NB. With NB and chemotherapy are the key factors for the favorable neurodevelopmental prognosis of OMS patients.

## Data Availability

The raw data supporting the conclusions of this article will be made available by the authors, without undue reservation.
